# Mitochondrial Biogenesis, Mitochondrial Dynamics, and Mitophagy in the Maturation of Cardiomyocytes

**DOI:** 10.3390/cells10092463

**Published:** 2021-09-18

**Authors:** Qianqian Ding, Yanxiang Qi, Suk-Ying Tsang

**Affiliations:** 1 School of Life Sciences, The Chinese University of Hong Kong, Hong Kong, China; 1155085756@link.cuhk.edu.hk; 2 State Key Laboratory of Agrobiotechnology, The Chinese University of Hong Kong, Hong Kong, China; yanxiangqi@link.cuhk.edu.hk; 3 Key Laboratory for Regenerative Medicine, Ministry of Education, The Chinese University of Hong Kong, Hong Kong, China; 4 The Institute for Tissue Engineering and Regenerative Medicine (iTERM), The Chinese University of Hong Kong, Hong Kong, China

**Keywords:** pluripotent stem cells, cardiomyocytes, mitochondria, mitochondrial biogenesis, mitophagy, mitochondrial dynamics, maturation

## Abstract

Pluripotent stem cells (PSCs) can undergo unlimited self-renewal and can differentiate into all the cell types present in our body, including cardiomyocytes. Therefore, PSCs can be an excellent source of cardiomyocytes for future regenerative medicine and medical research studies. However, cardiomyocytes obtained from PSC differentiation culture are regarded as immature structurally, electrophysiologically, metabolically, and functionally. Mitochondria are organelles responsible for various cellular functions such as energy metabolism, different catabolic and anabolic processes, calcium fluxes, and various signaling pathways. Cells can respond to cellular needs to increase the mitochondrial mass by mitochondrial biogenesis. On the other hand, cells can also degrade mitochondria through mitophagy. Mitochondria are also dynamic organelles that undergo continuous fusion and fission events. In this review, we aim to summarize previous findings on the changes of mitochondrial biogenesis, mitophagy, and mitochondrial dynamics during the maturation of cardiomyocytes. In addition, we intend to summarize whether changes in these processes would affect the maturation of cardiomyocytes. Lastly, we aim to discuss unanswered questions in the field and to provide insights for the possible strategies of enhancing the maturation of PSC-derived cardiomyocytes.

## 1. Process of Cardiomyocyte Differentiation and Maturation

The mammalian heart is the first differentiated and functional organ in the developing embryo. The heart originates from the cells of the early embryonic mesoderm, which emerges from the primitive streak during gastrulation. The myocardial progenitor cells migrate from the primitive streak to the anterior of the embryo and form the cardiac crescent. The early cardiac tube forms through the fusion of the cardiac crescent at the midline. The looping of the cardiac tube and expansion of the myocardium further leads to the formation of recognizable cardiac chambers [1,2]. The mammalian heart is composed of several major cell types: cardiomyocytes (CMs), smooth muscle cells, endothelial cells, as well as fibroblasts [3]. During cardiac development, there are two sources of cardiac progenitor cells (CPCs), namely the first heart field (FHF) and the second heart field (SHF) [4,5]. The CPCs of the FHF give rise to the left ventricle, whereas the CPCs of the SHF develop into a large part of the definitive heart including atria and inflow tract at the venous pole and right ventricle and outflow tract at the arterial pole [1,6,7].

The heart undergoes a complex differentiation and maturation process throughout embryonic and postnatal stages. The growth of the embryonic heart is mainly due to CM proliferation, while postnatal CMs lose the ability to proliferate soon after birth, and the continued increase in the heart mass is driven by the enlargement of existing CMs [8,9,10]. The differentiation and morphogenesis of mammalian hearts have been focal points for developmental cardiology. However, the maturation of the heart has been less studied until recently. Maturation is the last phase of heart development, a process in which immature CMs transit to mature CMs in terms of cell structure, gene expression, electrophysiological properties, and energy metabolism.

**Figure 1:**
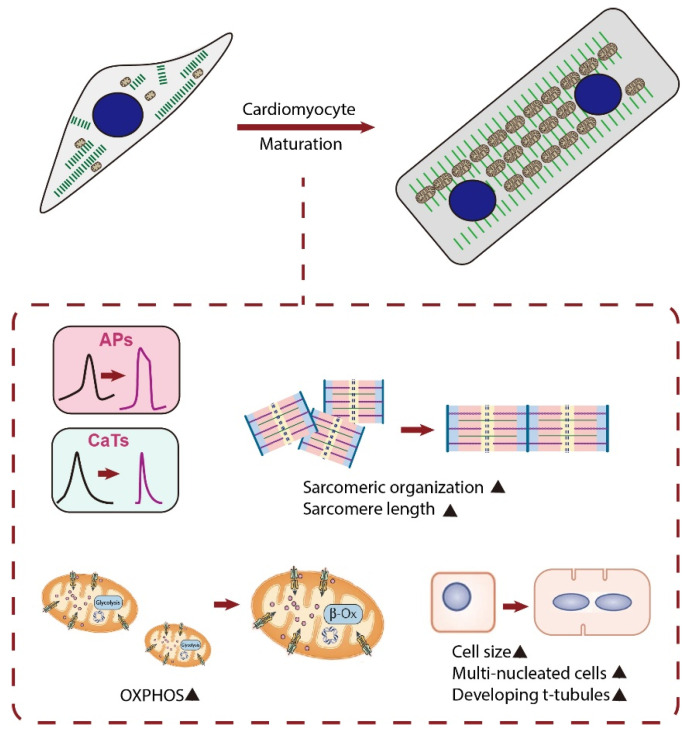
Schematic diagram showing major changes in characteristics of CMs during maturation. Matured CMs have an increased cell size, and the cell shape changes from circular to rod-shaped. Matured CMs have an improved alignment of sarcomeres, longer sarcomeres, and existence of t-tubules and become multi-nucleated. They also have enhanced calcium handling, electrophysiology, and metabolism. APs, action potentials; CaTs, calcium transients; OXPHOS, oxidative phosphorylation; β-Ox, β-oxidation.

The reason for the increased attention towards the maturation of CMs is the emerging pluripotent stem cell (PSC)-based regenerative therapy. Since the loss of CMs after heart injury is irreversible, PSC-derived CMs (PSC-CMs) have shown great promise in heart repair and functional improvement [11]. Although methods to differentiate PSCs into CMs have been well-established and those PSC-CMs show similar molecular, electrical, mechanical, and ultrastructural features to CMs, their fetal-like phenotypes limit the successful application of these cells for research and medicine [12,13]. Indeed, the following changes occur as CMs mature ex vivo: (i) adult cardiac genes instead of fetal cardiac genes are expressed; (ii) cell size increases and cell shape changes from circular to rod shape; (iii) sarcomere length increases and t-tubules are formed; (iv) electrophysiologically, action potential (AP) upstroke becomes faster, AP duration increases, maximum diastolic potential becomes more hyperpolarized, and diastolic depolarization slope decreases until the cells become electrically quiescent yet excitable; (v) mitochondria shape changes from small and round to firstly slender and long and subsequently ovular; (vi) metabolically, cells rely on oxidative phosphorylation (OXPHOS) instead of glycolysis for ATP production; and (vii) metabolic substrate changes from glucose to fatty acid [14,15] (Figure 1). However, PSC-CMs cultured under the conventional protocol are known to be largely immature with regard to the above features [14,15]. Although previous studies have tried to enhance PSC-CM maturation using different strategies, PSC-CMs are still regarded as unlike adult CMs. Specifically for mitochondria, mitochondria in PSC-CMs occupy a smaller cellular volume than that in adult CMs. Moreover, PSC-CMs rely on glycolysis rather than fatty acid β-oxidation (FAO) for ATP generation [16,17]. Thus, the challenge in front of us is to develop strategies to reach a higher degree of PSC-CM maturation in vitro.

## 2. Introduction to Mitochondria

Mitochondria are membrane-bound organelles found in the cytoplasm of eukaryotic cells. Mitochondria are morphologically heterogeneous among different cell types, varying from spherical-shaped, rod-shaped to a network [18]. The mitochondrion is enclosed by two membranes: the outer mitochondrial membrane (OMM) and inner mitochondrial membrane (IMM). The OMM is freely permeable to small molecules, while the IMM is where OXPHOS takes place and contains a variety of enzymes and transport proteins. The IMM is folded to form cristae to maximize cellular respiration output. An electrochemical membrane potential of about 180 mV generated by proton pumps builds up across the IMM [19,20]. The region between the two membranes is the intermembrane space. Inside the IMM is the mitochondrial matrix, which contains ribosomes, enzymes, and mitochondrial DNA (mtDNA) [21]. Mitochondria are regarded as semi-autonomous organelles since they are endowed with their genome and the complex machinery required for transcription, translation, and protein formation [22].

Mitochondria play a central role in the life and death of cells [23]. The most well-known function of mitochondria is energy production; mitochondria are responsible for cellular respiration and the production of most ATP in the cell. Respiration can be divided into three main pathways: glycolysis, the mitochondrial citric acid cycle, and mitochondrial electron transport [24]. The glycolytic pathway, the oxidization of glucose to pyruvate, takes place in the cytosol. The citric acid cycle (also known as the tricarboxylic acid cycle—TCA cycle) and electron transport occur within mitochondria. Pyruvate, the end product of glycolysis, enters mitochondria and is converted into acetyl coenzyme A (acetyl-CoA). Acetyl-CoA then reacts with oxaloacetate to start the TCA cycle and forms citrate. After the formation of citrate, a series of enzyme-catalyzed steps continues to occur in the TCA cycle, with the formation of aconitate, α-ketoglutarate, succinate, fumarate, malate, and ending in oxaloacetate [25,26]. The addition of the acetyl group to oxaloacetate forms citrate, and the cycle repeats. This process releases two CO_2_ molecules, an ATP molecule, and reducing equivalents, reduced nicotinamide adenine dinucleotide (NADH) and reduced flavin adenine dinucleotide (FADH_2_). NADH and FADH_2_ are then transported to the electron transport chain (ETC) and synthesize ATP by OXPHOS [27].

The ETC is made up of five multi-protein complexes (I to IV) that transfer electrons through the inner membrane of mitochondria to form a gradient of protons that drives the creation of ATP [24]. The electrons begin their reactions in complex I, continuing onto complex II, traversing to complex III and cytochrome c, and finally to complex IV. The ETC produces a higher concentration of H^+^ outside the inner membrane. As H^+^ ions flow down their gradient and back into the matrix, they pass through ATP synthase where ADP is converted into ATP [28].

Mitochondria are not merely the center for energy metabolism but are also the headquarters for different catabolic and anabolic processes, calcium fluxes, and various signaling pathways [29]. Mitochondria can sequester a large amount of Ca^2+^ from the cytoplasm and modulate the time course and amplitude of Ca^2+^ signals, working as a Ca^2+^ buffer system to maintain the Ca^2+^ homeostasis [30]. Mitochondrial Ca^2+^ uptake is driven by mitochondrial membrane potential (ΔΨ) and mediated by the mitochondrial calcium uniporter (MCU), a macromolecular structure that guarantees Ca^2+^ accumulation inside the mitochondrial matrix upon increases in cytosolic Ca^2+^ while Ca^2+^ release is under the control of the mitochondrial sodium-calcium exchanger (NCLX) and an H^+^/Ca^2+^ antiporter [31,32]. Ca^2+^ accumulation by mitochondria is a key component in the regulation of three essential dehydrogenases (pyruvate, α-ketoglutarate, and isocitrate dehydrogenase) that are the rate-limiting enzymes in feeding electrons at complex I of the respiratory chain [33]. An increase in the mitochondrial Ca^2+^ concentration thus results in activation of these three enzymes and an increase in ATP synthesis. However, large amounts of mitochondrial Ca^2+^ coinciding with oxidative stress may open the mitochondrial permeability transition pore (mPTP) [34], leading to the release of cytochrome c with subsequent initiation of apoptosis [35,36,37,38].

## 3. Mitochondrial Biogenesis

Mitochondrial biogenesis is the process by which cells increase mitochondrial mass. It was first described by John Holloszy in the 1960s, when it was discovered that physical endurance training induced higher mitochondrial content levels, leading to greater glucose uptake by muscles [39]. The mitochondrion is a unique organelle that contains its self-replicating genome. The mtDNA encodes 13 essential components of the ETC along with mitochondrial rRNA and tRNA [40]. However, the majority of mitochondrial proteins are encoded by the nuclear DNA and are transported to mitochondria after synthesis. Therefore, the biogenesis of mitochondria requires extensive coordination of both mitochondrial and nuclear genomes. Mitochondrial biogenesis is influenced by a variety of exogenous and endogenous factors such as exercise, caloric restriction, low temperature, oxidative stress, cell division, renewal, and differentiation [41]. The enhanced mitochondrial biogenesis increases the copy number of mtDNA, protein subunits of the metabolic enzymes, and ultimately result in a greater metabolic capacity.

A complex transcriptional network orchestrates nuclear and mitochondrial genome transcription and replication to conduct robust and dynamic mitochondrial biogenic responses. The expression of mitochondrial proteins encoded in the nuclear genome is regulated by transcription factors and transcriptional coactivators. The transcriptional coactivators related to mitochondrial biogenesis are the peroxisome proliferator-activated receptor γ-coactivator-1 (PGC-1) family, PGC-1α and PGC-1β. In mammals, mitochondrial biogenesis is primarily regulated by the transcriptional coactivator PGC-1α [42]. The most prevalent transcription factors activating promoters of mitochondrial genes are the nuclear respiratory factor 1 (NRF-1), nuclear respiratory factor 2 (NRF-2), and the estrogen-related receptor (ERR) family [43,44]. NRF1 and NRF2 are important contributors to the expression of subunits of respiratory complexes and mitochondrial transcription factors, including mitochondrial transcription factor A (TFAM), transcription factor B1 (TFB1M), and transcription factor B2 (TFB2M) [45,46]. ERRs are orphan nuclear receptors that serve a central function in the PGC-1 regulatory circuitry [47]. The ERR family (ERRα, ERRβ, and ERRγ) target vast gene networks involved in all aspects of energy homeostasis, including fatty acid and glucose metabolism, as well as mitochondrial biogenesis and function [48]. PGC-1β shares a similar molecular structure and function to PGC-1α, including nuclear-receptor binding and transcriptional activation, as well as regulating mitochondrial biogenesis. Unlike PGC-1α, PGC-1β is not upregulated in brown adipose tissue in response to cold and in muscle in response to exercise [49]. Other studies showed that PGC-1α can interact with both NRFs and ERRα, while PGC-1β interacts with NRF1, ERRα, ERRβ, and ERRγ [50,51,52]. While the orchestrated control of mitochondrial biogenesis is achieved largely through factors encoded by the nuclear genome, mitochondria-related mechanisms, such as mitochondrial protein import, mtDNA replication, transcription, and translation, also have an indispensable role in mitochondrial biogenesis [53]. In mammals, mitochondrial transcription requires the transcription factors TFAM and TFB2M, as well as mitochondrial RNA polymerase [54].

## 4. Mitophagy

Autophagy is the catabolic process through which cells degrade proteins and organelles with a double membrane structure, called autophagosomes. It is termed mitophagy when autophagosomes selectively sequester and degrade mitochondria [55]. Together with mitochondrial biogenesis, mitophagy establishes the balance to preserve mitochondrial homeostasis [56]. A regulated mechanism that eliminates dysfunctional mitochondria on purpose is essential for cells to maintain an appropriate bioenergetic system. In the IMM, ATP is synthesized through OXPHOS [57]. At the site of OXPHOS, mitochondria generate ATP as well as reactive oxygen species (ROS) such as superoxide (O_2_^−^), hydrogen peroxide (H_2_O_2_), and the hydroxyl free radical (·OH) [58,59,60]. Leakage of electrons from ETC is known to contribute to the generation of ROS [61]. It is well accepted that ROS leads to oxidative DNA damage, gene mutation, and diseases [62]. Since mtDNA has no protection from histones and its repair mechanisms are relatively weak, mtDNA is more vulnerable to oxidative DNA damage [63,64,65,66]. Accumulation of ROS in the mitochondrial matrix may increase the chance of mutation in mtDNA. Mutation in OXPHOS related genes results in the generation of faulty mitochondria, which could lead to ATP depletion, increased ROS production, and release of proapoptotic proteins [67,68,69]. Thus, mitophagy serves as an important biological process for mitochondrial quality control.

Mitophagy has been known to play a protective role in cells. Under stress conditions such as hypoxia, excessive ROS production, or nutrient deprivation, mitophagy can be induced. For instance, it has been reported that in mouse embryonic fibroblasts, under hypoxic conditions and therefore increased ROS production, hypoxia inducible factor-1-induced mitophagy is utilized to maintain redox homeostasis and thereby survival [70]. Similarly, in ischemia-reperfusion injury in neurons [71,72,73] and in the acute phase of myocardial infarction [74] in which excess ROS is produced, mitophagy has been reported to be responsible for removal of damaged and excessively aggregated mitochondria to prevent further injury.

On the other hand, mitophagy is also required in cellular metabolic transitions. ATP as a form of energy is mainly synthesized by OXPHOS and glycolysis. Cells may predominantly rely on one of them at a given stable state. Metabolic transitions are essential for cells facing different surrounding environments and their own intrinsic needs. Metabolic transitions are critical when cells are undergoing physiological or pathological processes such as differentiation, dedifferentiation, maturation, tumorigenesis, and metastasis. During metabolic transitions, existing mitochondria need to be replaced with a new set of mitochondria possessing appropriate metabolic capacity. By doing so, cells achieve the metabolic shift from a highly glycolytic state to highly OXPHOS state, or the reverse, depending on the cellular demand. A certain dominant metabolic profile is necessary for cells to maintain their physiological function. For example, Moussaieff et al. showed that early differentiation of human embryonic stem cells (hESCs) is accompanied by a switch of metabolic profile from more reliant on glycolysis to more reliant on OXPHOS [75]. Indeed, a number of studies have been conducted to demonstrate the involvement of mitophagy during multipotent stem cell differentiation, especially during metabolic remodeling. For instance, mitophagy has been shown to be necessary for the differentiation of myoblasts. It was found that glycolysis-favoring mitochondria in myoblasts are degraded through mitophagy immediately after the start of differentiation, and then a new population of mitochondria suitable for OXPHOS is generated, the suppression of which interferes with myogenic differentiation [76]. Programmed mitophagy is also needed to promote the metabolic switch from OXPHOS towards glycolysis during retinal ganglion cell differentiation from multipotent retinal progenitor cells [77]. Similarly, this process also contributes to removal of mitochondria during erythropoiesis [78]. Altogether, these results suggest that mitophagy could help in removing healthy mitochondria when metabolic transitions are needed.

Mitophagy might be conducted through several pathways. One of the necessary and conserved steps of the different pathways is the interaction between mitochondrial cargo receptors (MCRs) and microtubule-associated protein 1A/1B-light chain 3 (LC3)/GABARAP proteins, which link with autophagosomes. As more and more MCRs are identified, conserved LC3 interaction regions (LIRs) are required. As mentioned, mitophagy as a tool for mitochondrial quality control could selectively remove dysfunctional mitochondria. One of the important properties of damaged mitochondria is their depolarized membrane. To initiate mitophagy, dynamin-related protein (Drp1) helps to separate damaged mitochondria from the healthy population by mitochondrial fission [79,80]. PTEN-induced putative kinase-1 (PINK1) accumulates on the OMM of depolarized mitochondrion, where normally it is cleaved and digested [81]. PINK1 then phosphorylates Parkin E3 ligase, which ubiquitinates proteins at the OMM [82]. Phosphorylated mitofusin 2 (Mfn2) has been shown to be essential for the recruitment of Parkin [83]. MCRs including optineurin and nuclear dot protein 52 kDa then interact with ubiquitinated proteins and link them with LC3 [84,85]. Interestingly, a Parkin-independent mitophagy pathway that requires Drp1-mediated OMM severing and optineurin targeted ubiquitinated IMM was reported recently [86]. These results suggest that when the conventional Parkin-dependent pathway is blocked, cell-wide autophagy occurs as a substitute to remove damaged mitochondria [87]. Mitophagy may also rely on ubiquitin-independent pathways. BNIP3/BNIP3L(NIX) located at the OMM are stress-induced MCRs [88,89]. The LIR motif of BNIP3/NIX could directly bind to LC3/GABARAP proteins and recruit autophagosomes [90,91]. FUNDC1 also promotes hypoxia-induced mitophagy, but its interaction with LC3 proteins is highly regulated by its phosphorylation status. For instance, phosphorylation of the serine near its LIR motif (S17) was shown to be regulated by ULK1, a yeast ATG1 homologues [92]. Cardiolipin, a phospholipid of the IMM, has been shown to interact with LC3 in neurons and neuroblastic cells after moving to OMM [93].

## 5. Mitochondrial Dynamics

The cellular function of mitochondria is reflected in their structure. Mitochondria have a highly dynamic structure in a continuous balance between local fission and fusion, mediated by a cluster of dynamin-related guanosine triphosphatase (GTPase) proteins [94,95]. Mitochondrial fusion results in elongated, tubular, interconnected mitochondrial networks, while mitochondrial fission results in fragmented, discontinuous mitochondria. The fusion of mitochondria serves to mix and unify mitochondrial compartments. Fusion is beneficial by allowing minor defects in proteins or DNA of one mitochondrion to be complemented by functional proteins or DNA from another mitochondrion [96]. This is of particular importance for the inheritance and maintenance of the mitochondrial genome, which encodes several polypeptides required for respiratory function [97]. Fusion of the OMM is mediated by mitofusin 1 (Mfn1) and Mfn2, while fusion of the IMM is mediated by optic atrophy 1 (Opa1). Briefly, two mitochondria are tethered by Mfn1 and Mfn2, leading to the fusion of OMM. Then, the fusion of IMM occurs with the mediation of Opa1. Mitochondrial fission is specifically mediated by Drp1, which is a central component of the fission machinery [98]. When fission occurs, Drp1 translocates from the cytosol to the OMM where it interacts with receptor proteins, including fission protein-1 (Fis1), mitochondrial fission factor (Mff), and mitochondrial dynamics proteins of 49 (MiD49) and 51 kDa (MiD51) [99]. Then, Drp1 assembles into spirals and then constricts in a GTP-dependent manner, ligating and separating both IMM and OMM [100]. Drp1 constriction sites are often marked by ER, and the ER-mitochondrion contact occurs before and independently of Drp1 recruitment [101]. Mitochondria can undergo two kinds of fission: symmetrical and asymmetrical fission. Symmetrical fission occurs to replicate and expand the cellular mitochondrial pool [102,103], while asymmetrical fission is an initial step of a quality control mechanism [104]. During asymmetrical fission, damaged mitochondrial components are segregated from healthy components in an individual mitochondrion. The defective mitochondrion is then degraded and cleared by mitophagy [105].

Cells with a high energy demand such as the CMs and skeletal muscle cells tend to have a fused mitochondrial network, whereas those with low energy demand have a network where fission is more apparent. Accumulating evidence has demonstrated that mitochondrial dynamics are important for many aspects of cellular function, such as Ca^2+^ signaling [106], ROS production [107], mitophagy [104], embryonic development [108], and even lifespan [109].

Mitochondrial Ca^2+^ uptake controls a variety of cellular functions, such as aerobic metabolism, cytosolic Ca^2+^ signaling, and apoptosis. Drp-1-dependent mitochondrial fission was reported to block intraorganellar Ca^2+^ waves and protect against Ca^2+^-mediated apoptosis, indicating that mitochondrial dynamics control the spatiotemporal properties of mitochondrial and cellular Ca^2+^ and further affect the physiological and pathological status of the cell [106]. Interestingly, another study demonstrated that Ca^2+^ is involved in controlling mitochondrial morphology through intra-mitochondrial Ca^2+^ signaling. It was found that cells treated with ER Ca^2+^-ATPase inhibitor thapsigargin increased intracellular Ca^2+^ levels and mitochondrial Ca^2+^ influx, leading to mitochondrial fragmentation [110]. In CMs, increased cytoplasmic Ca^2+^-induced mitochondrial fission was reported to be required for CM hypertrophy [111]. All these data suggest that mitochondrial dynamics play crucial roles in cellular function through interaction with Ca^2+^.

As the main source of ROS production and key regulators for cellular redox signaling, mitochondria play a crucial role in ROS balance. It has been reported that inhibition of mitochondrial fission prevents Paraquat-induced ROS production and the subsequent apoptosis in mouse alveolar type II cells [112]. In beta-amyloid (Aβ)-induced neurodegeneration, there is a reciprocal relationship between mitochondrial dynamics and ROS in which accumulation of mitochondrial ROS triggers granular mitochondria formation, whereas inhibition of Drp1 reduces ROS level [113]. A study on human lung adenocarcinoma cells also showed that oxidative stress can induce mitochondrial fragmentation, and ROS scavengers prevented the fragmentation. However, the study indicated that neither Drp1 overexpression nor Mfn2 overexpression affected mitochondrial ROS generation in high-fluence low-power laser irradiated cells, indicating that mitochondrial fission was a relatively downstream event [114]. These studies provide evidence that mitochondrial ultrastructure is tightly coupled to ROS generation. However, the exact mechanism by which mitochondrial shape modulates mitochondrial function and redox homeostasis still needs more exploration.

Several studies have indicated that mitochondrial morphology changes during apoptosis, resulting in small, round, and more numerous organelles [103]. This process of fragmentation occurs early in the cell-death pathway, around the time that a proapoptotic member of the BCL2 family, BAX, translocates from the cytosol to mitochondria, but before caspase activation [115,116]. In adult CMs, although the mitochondria are packaged and confined among sarcomeres, mitochondrial physical communication is still active. In freshly isolated adult rat ventricular myocytes, the mitochondrial fusion is frequent, and weakened cardiac contractility by alcohol is associated with depressed mitochondrial fusion, indicating attenuated mitochondrial fusion might contribute to the pathogenesis of cardiomyopathy [117].

## 6. Mitochondrial Biogenesis and Cardiac Maturation

As we mentioned before, generating more physiologically mature CMs is vital for drug screening, disease modeling, and therapeutic purposes. As reported by many studies, structural and functional maturation of CMs is always accompanied by more mature energy metabolism. During early cardiac development, glycolysis is a major source of energy for CM proliferation. Perinatal mitochondrial biogenic surge in CMs is accompanied by a metabolic shift from using glucose to fatty acids for ATP generation [118]. As CMs mature and become terminally differentiated, mitochondrial oxidative capacity increases, with FAO becoming the major source of energy [119]. This shift is important to fulfill the increased cardiac workload as FAO is much more efficient than glycolysis for ATP generation. As development proceeds, mitochondria occupy ~20–40% of adult CM volume [120]. It has been documented that in PSC-CMs, the changes in energy metabolism have important impacts on the ability of CMs to proliferate during early cardiac development, as well as when CMs terminally differentiate during later development [121]. A previous study reported that an appropriate metabolic shift from aerobic glycolysis to OXPHOS would in turn improve metabolic and functional maturation of human PSC-CMs (hPSC-CMs) [122]. Fatty acid supplementation boosted hPSC-CM maturation with enhanced calcium transient peak height and kinetics and increased AP upstroke velocity and membrane capacitance [123]. Similarly, maturation media designed to provide oxidative substrates adapted to the metabolic needs of human induced pluripotent stem cell (hiPSC)-derived CMs (hiPSC-CMs) improved the physiological function of hiPSC-CMs [124].

As the key regulator of mitochondrial biogenesis and metabolism, PGC-1α is supposed to help promote cardiac maturation. PGC-1α knockout (KO) mice were viable, but postnatal growth of the heart and slow-twitch skeletal muscle, organs with high mitochondrial energy demands, is blunted [125]. PGC-1β KO mice exhibit phenotypes that are very similar to PGC-1α KO mice [126]. However, PGC-1α/β double-knockout (DKO) mice died shortly after birth with small hearts, bradycardia, intermittent heart block, and a markedly reduced cardiac output, suggesting the possibility that PGC-1α and PGC-1β control a subset of overlapping targets and are, therefore, capable of compensating for the loss of the other factor [127]. Cardiac-specific ablation of PGC-1α and PGC-1β caused a set of maturational defects including reduced growth, a late fetal arrest in mitochondrial biogenesis, and persistence of a fetal pattern of gene expression, indicating that PGC-1 is indispensable for perinatal maturation of the heart [127]. Recently, it has been demonstrated that PGC-1α can promote the maturation of CMs derived from hESCs [128]. The activator of PGC-1α, ZLN005, upregulated the expressions of PGC-1α and mitochondrial function-related genes in hESC-CMs and induced more mature energy metabolism compared with the control group. In addition, ZLN005 treatment increased cell sarcomere length, improved cell calcium handling, and enhanced intercellular connectivity [128]. Single-cell gene network analysis showed that PGC-1 drives CM maturation via YAP1 and SF3B2 [129]. All these data indicate that PGC-1 plays as a multifaceted regulator coordinating cellular hypertrophy, contractility, and metabolism of CMs from immature to mature.

PGC-1α and PGC-1β serve to coactivate downstream transcriptional events by interacting with specific transcription factors, including the ERRs. Conditional gene disruption strategies were used by Sakamoto et al. to detect the role of ERRs in cardiac differentiation and maturation. They demonstrated that ERRα and ERRγ are necessary for normal postnatal cardiac developmental maturation. ERRγ functions as a direct transcriptional activator of metabolic and structural cardiac genes. Moreover, ERRα/γ suppress a subset of fetal and non-cardiac myocyte genes, including the fibroblast lineage [130].

Apart from the predominant role of nuclear genes, mitochondrial genetic systems are also required for mitochondrial biogenesis. Among them, TFAM and TFB2M are reported to be required for mitochondrial genome replication and transcription [131]. A recent study showed that TFAM inactivation by the CRE-Lox system controlled under cardiac-specific Nkx2.5 locus caused mitochondrial dysfunction and embryonic lethal myocardial hypoplasia. Neonatal TFAM inactivation by AAV9-cTnT-Cre caused progressive, lethal dilated cardiomyopathy, while postnatal TFAM inactivation and disruption of mitochondrial function did not impair CM maturation [132]. The failure to observe CM maturation defects in postnatal TFAM inactivation is possible if TFAM functions only at an earlier stage before birth. However, more detailed investigation is needed to elucidate the exact role of TFAM and TFB2M in CM maturation.

**Figure 2:**
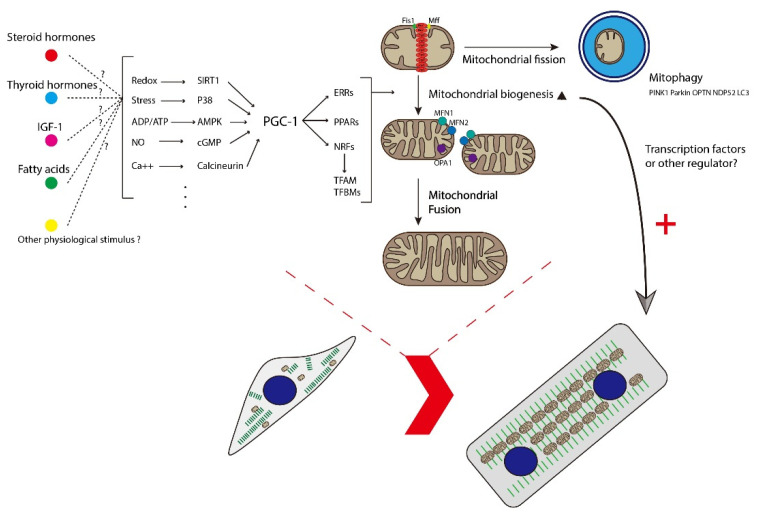
Schematic diagram showing how mitochondrial biogenesis, dynamics, and mitophagy participate in the maturation of CMs. Multiple stimuli activate PGC-1, leading to the coactivation of key transcription factors involved in several aspects of mitochondrial and cellular function. Mitochondria dynamically change their morphology through the cycle of fusion and fission. The main fusion factors are OPA1, MFN1, and MFN2, which bind to the IMM and OMM of mitochondria. Drp1 is a major fission factor that binds to OMM and forms a ring-like structure around mitochondria, leading to the separation of mitochondria into two. Mff and Fis1 function as adaptors to recruit Drp1 to the OMM. Mitochondrial fission and mitophagy function as quality control to segregate and degrade immature or damaged mitochondria and to provide materials for mitochondrial biogenesis. Healthy mitochondria tend to fuse together and are believed to function well. The maturation of healthy mitochondria further promotes the maturation of CMs at structural and functional levels. IGF-1, insulin-like growth factor 1; PGC-1, peroxisome proliferator-activated receptor γ coactivator 1; ERR, estrogen-related receptor; PPARs, peroxisome proliferator-activated receptors; NRF, nuclear respiratory factor; TFAM, transcription factor A, mitochondrial; TFBMs, mitochondrial transcription factor B; Fis1, fission protein-1; Mff, mitochondrial fission factor; OPA1, optic atrophy 1; MFN1, mitofusin 1; MFN2, mitofusin 2; PINK1, PTEN-induced kinase 1; OPTN, optineurin; NDP52, calcium binding and coiled-coil domain 2; LC3, microtubule-associated protein 1A/1B-light chain 3.

Taken together, these results showed that mitochondrial biogenesis is essential for CM maturation (Figure 2) and unveiled a new strategy to improve the maturation of PSC-CMs and therefore to generate more physiologically mature CMs for drug screening, disease modeling, and therapeutic purposes.

## 7. Mitophagy and Cardiac Maturation

After birth, the immature heart of a mammal is exposed to a different environment with relatively higher oxygen concentration and lower carbohydrate supplement [133,134]. Therefore, the metabolic transitions through which CMs switch to rely on FAO as primary energy source are needed [135]. This is not just an adaptative response; the growing organism requires a powerful, energy consuming heart with efficient ATP synthesis [136]. Immature fetal mitochondria showing few and sparse cristae for ETC are incapable of efficient ATP production; therefore, substitution or manipulation of mitochondria is needed. Although it has been conventionally believed that changes in mitochondrial gene expression at transcriptional level cause this transition [137], Gong et al. suggested that mitophagy-mediated replacement of fetal mitochondria is essential for this transition [138]. They interrupted Parkin-dependent mitophagy by generating transgenic mice with a mutant version of Mfn2 that can be temporally and spatially expressed in CMs starting from birth [138]. The mutant Mfn2 could inhibit mitochondrial translocation of Parkin but could still promote mitochondrial fusion as wild-type Mfn2 does [138]. Compared with mice expressing wild-type Mfn2, mutant Mfn2 mice persisted fetal mitochondrial morphology and immature mitochondrial function [138]. Perinatal mutant Mfn2 expression also failed the metabolic gene reprogramming and led to cardiomyopathy [138]. This study demonstrated the role of mitophagy during postnatal maturation of CMs. Since reliance on glycolysis as the primary energy source is one of the features of PSC-CMs, it is reasonable to investigate the role of mitophagy in the maturation of PSC-CMs [14]. Zhao et al. reported the investigation on mitophagy during maturation of ESC-CMs. They found that the promotional effect of glucocorticoids on the maturation of ESC-CMs may be related to Parkin-dependent mitophagy [139]. Interruption of mitophagy through both autophagy inhibition and knocking down of Parkin expression could block the promotional effect of glucocorticoid on ESC-CM maturation [139]. PINK1-Parkin-dependent mitophagy targeting depolarized mitochondria is thought to carry out quality control of mitochondria. The abovementioned evidence suggests that this pathway is not only a passive response after the appearance of dysfunctional mitochondria but also an available tool for programmed mitochondrial clearance. Thus, it might be reasonable to ask whether manipulating mitophagy could promote the maturation of CMs (Figure 2).

## 8. Mitochondrial Dynamics and Cardiac Maturation

Mitochondrial fission and fusion events transmit signaling messengers into changes of mitochondrial function, such as calcium buffering and metabolism within the cell. Mitochondrial dynamics have been implicated in a variety of biological processes including embryonic development. It has been proven by several groups that the deletion of proteins regulating mitochondrial dynamics, such as Mfn1, Mfn2, and Drp1, causes defects in cardiac development [140,141,142,143], suggesting that mitochondrial dynamics play vital roles in cardiogenesis.

To reveal the function of mitochondrial dynamics in cardiac development and maturation, researchers simultaneously ablated Mfn1 and Mfn2 in the embryonic or adult mice heart [140,144,145]. The study of Chen et al. showed that embryonic combined Mfn1/Mfn2 ablation was lethal after E9.5. Conditional combined Mfn1/Mfn2 ablation in adult hearts induced mitochondrial fragmentation, respiratory dysfunction, and rapidly progressive and lethal dilated cardiomyopathy [144]. Kasahara et al. also found that embryo lethality occurred after E9.5 in Mfn1/Mfn2 DKO mice; the DKO hearts were markedly hypoplastic, with biventricular wall thinning and poor trabeculation [140]. In addition, gene-trapping of Mfn2 or Opa1 in mouse ESCs impaired the differentiation of ESCs into CMs. The following mechanistic study showed that increased Ca^2+^-dependent calcineurin activity and Notch1 signaling might be responsible for the impaired ESC differentiation because fragmented mitochondria have decreased Ca^2+^ buffer ability, leading to increased cytosolic Ca^2+^ [140]. DKO mice with Mfn1 and Mfn2 being genetically inactivated in mid-gestation using a loxP/Myh6-cre approach were reported to have normal cardiac morphology and function at birth [145]. The mitochondria increased in number and appeared to be spherical and heterogeneous but exhibited normal electron density. By postnatal day 7, the mitochondrial number continued to increase, and many lost matrix components and membrane organization. The DKO mice developed dilated cardiomyopathy, and all died before postnatal day 16. Gene expression analysis showed that mitochondria biogenesis genes and mitophagy markers are altered, resulting in decreased mitochondrial biogenesis and hampered mitochondrial elimination [145]. All these studies indicate that mitochondrial fusion is essential for embryonic cardiac differentiation and postnatal cardiac development. In vitro study of hiPSC differentiation into CMs showed that enhanced mitochondrial fusion in hiPSCs significantly increased the expression of cardiac-specific genes, making mitochondrial fusion promoters promising molecular targets for generating lineages of the heart from hiPSCs for patient-specific regenerative medicine [146].

As the key factor of mitochondrial fission, the role of Drp1 in CM development was also determined. Drp1 was deleted by using a Myh6-Cre transgenic line [147]. Echocardiography at postnatal day 7 revealed that left ventricular function was significantly compromised, with decreased contraction and heart rate. All Drp1 KO mice died between postnatal day 9 and day 11. In addition, mitochondria interconnectivity increased, and respiration decreased in Drp1 KO CMs [148]. Similarly, muscle-specific Drp1 KO mice showed neonatal lethality due to dilated cardiomyopathy. The Drp1 ablation in heart and primary cultured CMs resulted in severe mtDNA nucleoid clustering and led to compromised mitochondrial respiration, which further led to immature myofibril assembly and defective CM hypertrophy [149]. On the contrary, the study of Hoque et al. demonstrated that changes in mitochondrial morphology from a small granular fragmented phenotype in PSCs to a filamentous reticular elongated network in differentiated CMs were detected during cardiac mesodermal differentiation and maturation. Interestingly, treatment of iPSCs with Mdivi-1, a pharmacological inhibitor of mitochondrial fission protein Drp1, during cardiac differentiation increased the percentage of beating embryoid bodies and expression of cardiac-specific genes. In addition, Drp1 gene silencing was accompanied by increased mitochondrial respiration and decreased aerobic glycolysis [150]. These results indicated that Drp1-mediated mitochondrial fission is indispensable for cardiac development, but a moderate shifting of mitochondrial morphology toward fusion by inhibition of Drp1 may help to promote cardiac differentiation and maturation with a metabolic shift from glycolysis towards OXPHOS, as fused and interconnected mitochondria are regarded to function well and exhibit enhanced metabolic capacity. Taken together, mitochondrial dynamics play important roles in cardiac development. Manipulating mitochondrial fission and fusion may be one of the possible ways to improve the maturation of CMs (Figure 2).

## 9. Discussion and Future Direction

Mitochondrial biogenesis and mitophagy together regulate the quantity and quality of mitochondrial network. To achieve this, actively operated mitochondrial fusion and fission are indispensable. We summarize here that mitochondrial biogenesis, mitophagy, and mitochondrial dynamics contribute to the successful specification and maturation of CMs.

Interestingly, mitochondrial metabolites from the TCA cycle (e.g., acetyl-Co A, citrate, aconitate, α-ketoglutarate, succinate, fumarate, malate, oxaloacetate) are reported to control unique cellular function and cell fate [151,152]. The fact that the metabolic process is regulated by mitochondrial metabolites, such as NADH/NAD+, ATP/ADP, ATP/AMP, and succinate/α-ketoglutarate, indicated a non-negligible role of mitochondrial metabolites [151,153,154,155]. For instance, recent studies showed that mitochondrial metabolites can regulate cell function through modulating gene transcription and translation in cancer cells and immune effector cells [156,157,158,159]. In addition, several TCA metabolites, such as acetyl-CoA and succinate, have been demonstrated to participate in cardiovascular diseases including diabetes and ischemia-reperfusion injury [160,161,162]. For their potential role in cell fate determination, it was reported that in naïve embryonic stem cells, glutamine-derived α-ketoglutarate helps maintain a high α-ketoglutarate-to-succinate ratio, which is important for promoting histone/DNA demethylation and for maintaining pluripotency [163]. In addition, α-ketoglutarate was found to promote early differentiation of PSCs [164,165]. However, while previous studies have demonstrated the involvement of metabolism in CM development and maturation as indicated in previous sections, to the best of our knowledge, the direct role of mitochondrial intermediate metabolites in CM development and maturation is unexplored. Further investigation is certainly needed for this interesting and important research direction.

In addition, the physiological stimuli that could promote CM maturation through regulating mitochondrial biogenesis are in need of further exploration. The transition of metabolic reliance towards FAO after birth and the surge of various hormones in or after late gestation should be noticed. For instance, observation of dramatic rise in glucocorticoid levels shortly before birth led to the discovery of PGC-1α’s role as a downstream target of the glucocorticoid receptor in glucocorticoid-promoted maturation of the fetal heart [166]. Further investigation along this direction is needed.

Moreover, the interaction of cardiac transcription factors with PGC-1 may be tested. Similarly, increasing in mitochondrial amount and mass might generate signals which regulate maturation of CMs. Some signals including AMP-to-ATP ratio and NAD+-to-NADH ratio change simultaneously with the change in mitochondrial functions. Signaling pathways including AMPK, STAT3, and SIRT3 have been shown to be related to the retrograde signaling from mitochondria to nuclei. The positive involvement of cardiac transcription factors in these retrograde signaling pathways requires more detailed studies.

The role of Parkin-mediated mitophagy in regulating metabolic maturation during postnatal development of mouse heart has been revealed. However, this knowledge has not been sufficiently discussed or applied in the issue of PSC-CM immaturity. More attention should be paid to identifying different pathways of mitophagy in CMs and their potential contribution to promote maturation of PSC-CMs. In addition, a deeper investigation of upstream signaling, which may regulate mitophagy, may lead to a method to enhance the maturation of PSC-CMs.
